# Analysis of AM Parameters on Surface Roughness Obtained in PLA Parts Printed with FFF Technology

**DOI:** 10.3390/polym13142384

**Published:** 2021-07-20

**Authors:** Irene Buj-Corral, Xavier Sánchez-Casas, Carmelo J. Luis-Pérez

**Affiliations:** 1 Department of Mechanical Engineering, Barcelona School of Industrial Engineering (ETSEIB), Universitat Politècnica de Catalunya-Barcelona Tech (UPC), 08028 Barcelona, Spain; xasc95@gmail.com; 2 Engineering Department, Public University of Navarre (UPNA), Arrosadia Campus, 31006 Pamplona, Spain; cluis.perez@unavarra.es

**Keywords:** additive manufacturing, surface roughness, FFF, ANFIS, modeling

## Abstract

Fused filament fabrication (FFF) 3D printing technology allows very complex parts to be obtained at a relatively low cost and in reduced manufacturing times. In the present work, the effect of main 3D printing parameters on roughness obtained in curved surfaces is addressed. Polylactic acid (PLA) hemispherical cups were printed with a shape similar to that of the acetabular part of the hip prostheses. Different experiments were performed according to a factorial design of experiments, with nozzle diameter, temperature, layer height, print speed and extrusion multiplier as variables. Different roughness parameters were measured—*Ra*,* Rz*,* Rku*,* Rsk*—both on the outer surface and on the inner surface of the parts. Arithmetical mean roughness value Ra and greatest height of the roughness profile *Rz* are usually employed to compare the surface finish among different manufacturing processes. However, they do not provide information about the shape of the roughness profile. For this purpose, in the present work kurtosis *Rku* and skewness *Rsk* were used. If the height distribution in a roughness profile follows a normal law, the *Rku* parameter will take a value of 3. If the profile distribution is symmetrical, the *Rsk* parameter will take a value of 0. Adaptive neural fuzzy inference system (ANFIS) models were obtained for each response. Such models are often employed to model different manufacturing processes, but their use has not yet been extended to 3D printing processes. All roughness parameters studied depended mainly on layer height, followed by nozzle diameter. In the present work, as a general trend, *Rsk* was close to but lower than 0, while *Rku* was slightly lower than 3. This corresponds to slightly higher valleys than peaks, with a rounded height distribution to some degree.

## 1. Introduction

Over the last two decades there has been a huge increase in the demand for orthopedic implants due to an aging population and increasing life expectancy [1]. In the specific case of hip arthroplasty, this has increased by 30% worldwide compared with the year 2000 [2]. At the State level, there has also been an increase in this type of surgery, from 72 interventions per 100,000 inhabitants in 2000 to 102 per 100,000 inhabitants in 2011, which represents a total of 47,006 implants that year [1]. Across Europe, 650,000 hip prostheses were implanted in 2017, and growth is estimated at 2% per year until 2024, when it is expected to exceed 730,000 implants [3]. This increase in demand has prompted the improvement of biomaterials, manufacturing methods and design processes of these medical devices.

Materials used for medical applications must be biocompatible, which means that not only must they not be toxic, but the interaction of the material with the cells must have a positive effect. In this way, most of the prostheses used today are either made of metal or by a combination of metal with plastic parts. In addition, its design must mimic the morphological structure of bones and promote osteogenesis, that is, the formation of bone tissue. Historically, the fixation of these implants has been achieved using polymethylmethacrylate (PMMA), an acrylic polymer commonly known as “bone cement”. However, this fixation method carries the risk of the bone cement degrading over time, and it may come off or cause inflammation in adjacent tissues [4]. From the 1980s onwards, non-cemented prostheses appeared on the market, which, unlike cemented ones, are biologically fixed to the host bone. This fixation is achieved by making prostheses with a porous coating so that bone tissue can regenerate by infiltrating the porosity of the implant, a phenomenon called osseointegration.

In recent years, different additive manufacturing (AM) processes have been used to manufacture orthopedic implants. Specifically, extrusion 3D printing processes provide parts with complex shapes and, specifically, with porous structures, to be obtained [5]. In addition, extrusion 3D printing processes do not require the use of molds or dies, thus allowing the manufacture of customized prostheses at relatively low cost [6]. Polylactic acid (PLA) is a biocompatible material used in orthopedic applications [7]. Unlike other plastic materials such as polyglycolide (PGA), PLA has a low degradation rate and, for this reason, inflammatory response occurs as late as 2 or 3 years after the operation [8]. Nonetheless, in a study on implanted pins, rods, bolts and screws, PLA showed a low incidence of adverse tissue reaction of 0.2% [9].

Several authors have studied the influence of 3D printing parameters on average surface roughness Ra of the lateral walls obtained in fused deposition modeling (FDM) processes, also known as fused filament fabrication (FFF). For instance, Rahman et al. [10] found that low bed temperature, high 3D printing speed, medium infill, low layer thickness and a low number of shells are recommended in order to reduce Ra (arithmetical mean roughness value) when 3D printing acrylonitrile-butadiene-styrene (ABS) components. Peng and Yan [11] studied PLA parts and found that lowest Ra and Rz (greatest height of the roughness profile) values were obtained with low layer height and high speed. In addition, roughness is greatly influenced by the measurement direction. The higher the inclination of a wall is, the higher the theoretical value of the Ra parameter becomes [12,13]. Regarding other roughness parameters, in a previous study with PLA cylindrical parts, it was found that, as a general trend, slightly negative Rsk (skewness) values are obtained in lateral walls of printed parts, corresponding to slightly sharp valleys and rounded peaks. The Rku (kurtosis) values ranged between 1.7 and 2.3 for different 3D printing angles [13]. Alsoufi and Elsayed also reported negative values for Rsk, with higher Rku between 2.7 and 3.4 for PLA [14]. For reinforced PLA, Rku values increased up to almost 6. All measurements correspond to flat vertical walls. However, few studies are known about kurtosis and skewness on curved surfaces of 3D printed parts.

On the other hand, over the last few years fuzzy inference systems (FIS) and adaptive neural fuzzy inference systems (ANFIS) have been widely employed for modelling output variables in manufacturing processes, where Takagi–Sugeno [15] and Mamdani [16,17] are the most commonly FISs employed. Several studies can be found in the literature dealing with the application of design of experiments (DOE) along with conventional statistical regression and soft computing techniques in order to analyze the influence of process parameters over some response variables in manufacturing engineering. In this way, hybrid learning procedures that combine artificial neural networks (ANNs) and fuzzy inference systems (FISs), which are named as adaptive-neural fuzzy inference systems (ANFIS), are one of the most interesting techniques for this purpose. From the research study of Jang [18], several studies have been developed that employ ANFISs, as can be observed in the review of the state of the art by Shihabudheen and Pillai [19]. Some other examples found in the literature are that of Aamir et al. [20], which used a Mamdani FIS in order to predict the surface roughness and hole size as a function of feed rate and cutting speed in multi-hole drilling; that of Pandiyan et al. [21], where the authors employed a Sugeno FIS and a Taguchi experimental design as the inputs for the ANFIS model in order to predict the material depth of cut [21]; and that of Mensah et al. [22], which employed an adaptive neuro-fuzzy inference system to predict both the heat release capacity and the total heat release of the extruded polystyrene measured from microscale combustion calorimetry experiments using a Sugeno FIS—where these authors found that the ANFIS can be used accurately and reliably in flammability studies, among the many other research studies that could be mentioned.

With regard to additive manufacturing, in recent years, the application of machine learning and soft computing techniques are gaining a growing interest [23], as can also be observed in studies such as that of Saleh et al. [24], which analyzed the effect of water-silica slurry impacts on polylactic acid (PLA), processed by fused deposition modelling (FDM), by using an ANFIS in which building orientation, layer thickness, and slurry impact angle were the inputs and weight gain resulting from water, net weight gain, and total weight gain were the outputs. In their study, a Taguchi orthogonal array was used for planning the experiments. Moreover, generalized bell membership functions and a Sugeno FIS were also considered in their study. Among their conclusions, the authors found that the ANFIS could adequately predict the effect of slurry impacts on PLA material processed by FDM [24]. Likewise, Kumar et al. [25] employed a fuzzy inference system combined with the Taguchi philosophy for optimization of the FDM process parameters. These authors employed a Mamdani FIS and triangular membership functions. Moreover, they used the centroid in order to de-fuzzify the aggregated output. Some other studies, such as that of Sai et al. [26], employed a central composite design and an ANFIS to analyze the influence of process parameters in FDM of PLA implants. Moreover, hybrid optimization techniques based on genetic algorithm-adaptive neuro fuzzy interface system (GA-ANFIS) have also been used in order to optimize the FDM process parameters, as can be observed in Deshwal et al. [27]. Another study developed by Rajpurohit et al. [28] employed an ANFIS for prediction of tensile strength in FDM parts. Some other research studies worth mentioning are that of Mahesh et al. [29], in which an FIS is employed for decision and benchmarking in order to select appropriate rapid prototyping and manufacturing processes, such as SLA, SLS and FDM, among other processes; that of Yadav et al. [30], in which the influence of layer height, material density and extrusion temperature on the mechanical properties of FDM parts is analyzed by using an ANFIS; and the research study of Huynh et al. [31], in which a Taguchi design of experiments along with a Mamdani FIS using triangular membership functions is employed to determine the accuracy of manufactured PLA parts using FDM. Regression models are usually employed to analyze the effect of printing parameters on roughness in FDM processes [32,33].

In the present work, hemispherical cups were printed in PLA material with the FFF extrusion technology. Different roughness parameters such as Ra, Rz, Rsk and Rku were measured, by means of a contact roughness meter. The first two parameters are usually employed for comparison of manufactured surfaces. The last two mentioned parameters give information about the shape of the roughness profile. Roughness was determined on both the external and the internal surfaces of the specimens in order to assess if there are differences in the surface finish of both surfaces. Those differences could be attributed to the solidification process of the material, mainly in contact with air and with the printing supports, respectively. ANFIS models were obtained for each response as a function of the process parameters, and the main variables influencing roughness were determined. The ANFIS models have the advantages of modeling nonlinear systems, high adaptation capability and a fast learning process [34]. The present work will help to select appropriate printing processes when curved surfaces are to be 3D printed in PLA. Moreover, the results could be applied to other extrusion 3D printing processes such as direct ink writing (DIW) for inert ceramics, which can also be employed to manufacture prostheses.

## 2. Materials and Methods

### 2.1. D printing Process

**Figure 1:**
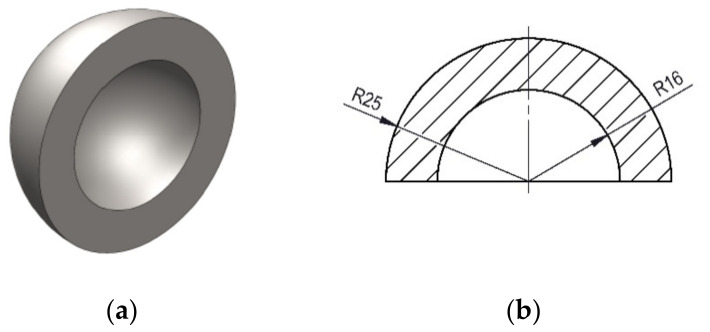
3D-printed hemispherical cups (**a**) shape; (**b**) dimensions.

Hemispherical cups were printed of internal dimeter 32 mm and external diameter 50 mm, according to Meftah et al. [35] (Figure 1).

**Figure 2:**
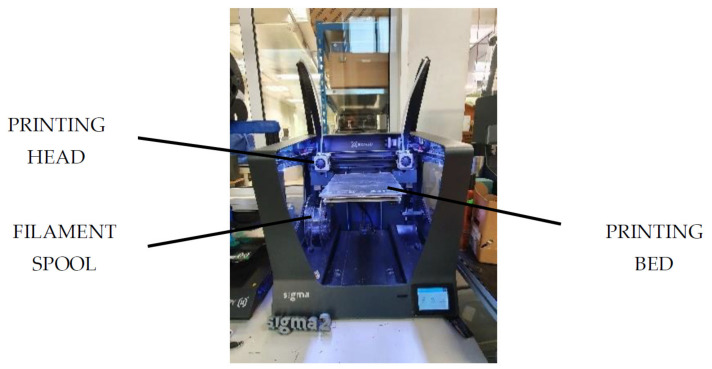
Sigma R19 from BCN3D.

White polylactic acid (PLA) filament of 2.85 mm diameter from BCN3D was used to print the samples in a SigmaR19 printer from BCN3D Technologies, Castelldefels, Barcelona, Spain (Figure 2).

Fixed 3D printing parameters were infill 20%, shell thickness of 1.2 mm, shell pattern concentric, infill pattern grid, printing bed temperature 65 °C. Printing supports were required. They were printed in PLA and removed by hand. Infill of the support was 50%, the grid pattern was used, z offset = 0, and xy offset = 0. The maximum angle without supports was fixed as 30°. The experiments were defined according to a fractional factorial design 2^5−1^, with 5 variables and 2 levels. The five selected variables presented in Table 1 have been shown to influence roughness. Their values were selected according to the machine manufacturer’s recommendations for PLA, as well as from the results of previous studies [13]. The infill value was set to 20% so that the printing time was low.

The selected output values are different roughness parameters as shown in Table 2.

Ra and Rz parameters are widely used to characterize the surface finish of manufactured parts. However, those parameters do not provide information about the shape of the roughness profile. For example, they cannot discriminate between surfaces with high or low interference shear strength. For this purpose, other parameters such as kurtosis Rku and skewness Rsk are recommended [36]. Kurtosis corresponds to the peakedness of the profile, while skewness is related to its symmetry. Ideally, Rku should be close to 3, corresponding to a normal distribution of heights in the profile, and Rsk should be 0 in order to obtain a symmetrical profile. However, in extrusion 3D printing processes Rku values below 2 and slightly negative Rsk values have been reported [37], corresponding to highest valleys than peaks and a slightly rounded profile.

### 2.2. Roughness Measurement

Roughness was measured with a Talysurf 2 contact roughness meter from Taylor Hobson Ltd, Leicester, UK. A diamond tip was used with tip angle of 90° and tip radius of 2 µm. Measuring force is 0.8 mN and speed is 0.5 mm/s. A Gaussian filter was employed. A cut-off value of 0.8 mm was used according to ISO 4288 [38]. Total sampling length was 4.8 mm (6 × 0.8 mm).

Roughness was measured along generatrices of both the external and the internal surfaces of the hemispherical cups, in order to assess if there were differences regarding their surface finish. The internal surface of the cup is concave, and it solidifies on a printing support (the printing position is shown in Figure 1b). This is expected to produce heat transfer by means of conduction with the support and between adjacent filaments, radiation between adjacent filaments, and convection with entrapped air, with low refrigeration. On the contrary, the external surface of the cup is convex, and it is expected to solidify in contact with air, mainly because of convection and radiation with the environment [39]. For this reason, the external surface of the cups is likely to solidify more quickly than the internal one.

### 2.3. ANFIS Modelling

A zero-order Sugeno FIS was employed by using the Fuzzy Logic Toolbox™ of Matlab^TM^2020a [40], from Mathworks, Natick, MA, USA, because the de-fuzzification process for a Sugeno system is computationally more efficient compared with that of a Mamdani system [40,41,42,43]. An FIS with “if then” rules was built from the DOE, where the membership functions for fuzzification of the independent variables were Gaussian and are shown in Equation (1).

(1) $$\mu_{x}=e^{\frac{-{\left(x-c\right)}^{2}}{2\sigma^{2}}}$$

The aggregation method is the sum of fuzzy sets, and the aggregated output is obtained from the weighted average of all output rules. Equation (2) shows the implication method and Equation (3) shows the output of the Sugeno system [40,41,42,43].

(2) $$\lambda_{j}\left(x\right)=AndMethod\left\{\mu_{1}\left(x_{1}\right),\ldots ,\mu_{n}\left(x_{n}\right)\right\}$$

(3) $$\left\{Roughness\right\}=\frac{\sum_{j=1}^{Number\;of\;rules}\lambda_{j}\times z_{j}}{\sum_{j=1}^{Number\;of\;rules}\lambda_{j}}$$

**Figure 3:**
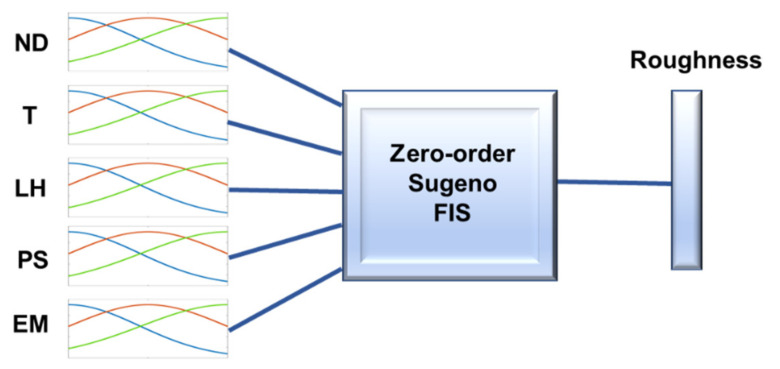
Gaussian membership functions employed to obtain the fuzzy inference system for modelling the roughness parameter (before the ANFIS).

From the above, a zero-order Sugeno FIS [40,41,42] was selected with Gaussian membership functions. Figure 3 shows the Gaussian membership functions for modelling the roughness parameters before using the ANFIS. In order to tune the so-generated fuzzy system, an ANFIS employing the Fuzzy Logic Toolbox of Matlab^TM^ along with a back-propagation algorithm was used [40].

The roughness parameters analyzed in this present study were the arithmetical mean roughness value or arithmetical mean of the absolute values of the profile deviations from the mean line of the roughness profile $\left(Ra\right)$, which is one of the most commonly employed parameters in industry; the mean roughness depth or average maximum peak to valley of five consecutive sampling lengths of the profile within a sampling length $\left(Rz\right)$; the kurtosis $\left(Rku\right),$ which is a measure of the sharpness of the profile; and the skewness $\left(Rsk\right),$ which measures the symmetry of the profile. These parameters are defined in the UNE-EN-ISO 4287:1999 standard [44]. Figure 3 depicts the Gaussian membership functions used to obtain the fuzzy interference system for modelling roughness.

**Figure 4:**
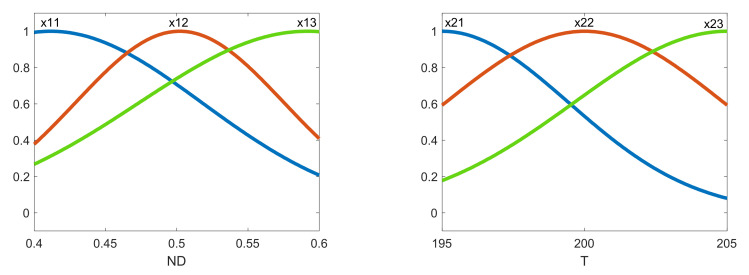

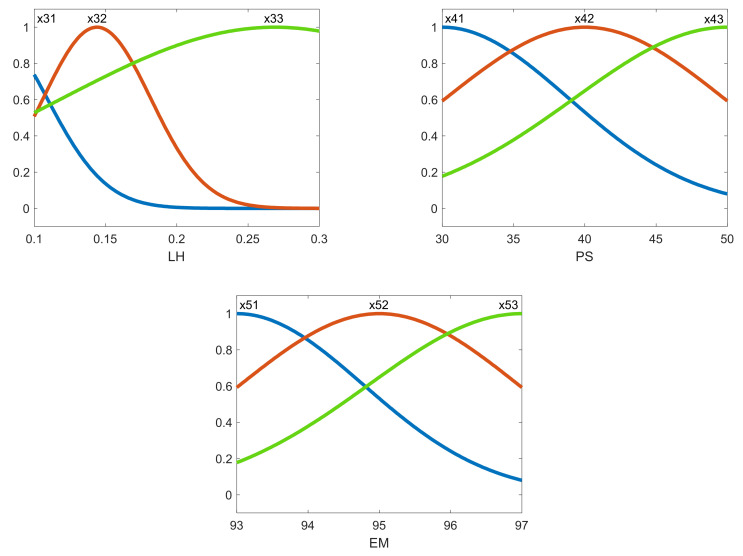
Membership functions obtained after using the ANFIS for the case of Ra measured on the outer layer of the manufactured prototypes.

Once the FIS was obtained, then the ANFIS tuned the membership parameters. Figure 4 shows the membership functions after the FIS for the case of the arithmetic average roughness of the roughness profile $\left(Ra\right)$ on the outer layer of the manufactured prototypes.

As shown in Figure 4, the membership functions were tuned after running the ANFIS, compared with those shown in Figure 3. For each of the response variables—*Ra*, *Rz*, *Rsk* and *Rku*—the obtained ANFIS was used to model the response variables.

## 3. Results and Discussion

### 3.1. Roughness

Table 3 presents the roughness results for both the internal and the external surfaces of the hemispherical cups. One measurement was performed on each surface.

For external roughness, lowest Ra and Rz values below 8 µm and 38 µm respectively corresponded to low layer height, low nozzle diameter and low temperature. Rku values ranged between 2.1 and 2.8, while Rsk values ranged between −0.773 and 0.123. Similar results were obtained for the internal and the external surfaces. Highest difference of 21% was observed for experiment 9. In another work with hollow prismatic specimens, similar difference values were reported [12].

**Figure 5:**
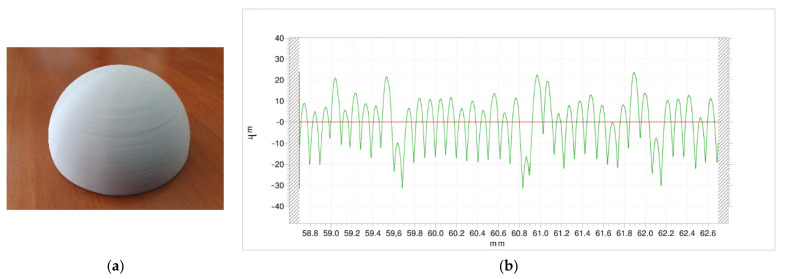
External surface of experiment 12, obtained with high nozzle diameter, low layer height, high print speed, high temperature and low extrusion multiplier: (**a**) Image, (**b**) Roughness profile.

Figure 5 shows the external surface of experiment 12, obtained with high nozzle diameter, high temperature, low layer height and high print speed. The characteristic stair-stepping effect of the FDM-3D printed parts is observed (Figure 5a). Ra value is 8.90 µm. In the profile (Figure 5b), peak height ranges between 5 µm and 22 µm, suggesting some displacement between layers.

**Figure 6:**
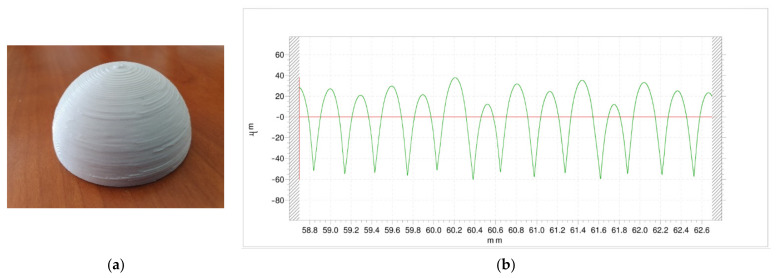
External surface of sample 16, obtained with high nozzle diameter, high layer height, high print speed, high temperature and high extrusion multiplier: (**a**) Image, (**b**) Roughness profile.

Figure 6 corresponds to the external surface of experiment 16, obtained with high nozzle diameter, high temperature, high layer height and high print speed. As a general trend, the combination of high nozzle diameter and high layer height is not recommended, not only because it provides high roughness values but also because an excess of material is observed in some areas of the surface (Figure 6a). However, roughness was measured in an area without protuberances. For this reason, a relatively steady profile was obtained (Figure 6b). Ra value was 21.77 µm. Peak height ranged between 10 µm and 40 µm, corresponding to the lateral misalignment of the layers.

### 3.2. Roughness Modelling

**Figure 7:**
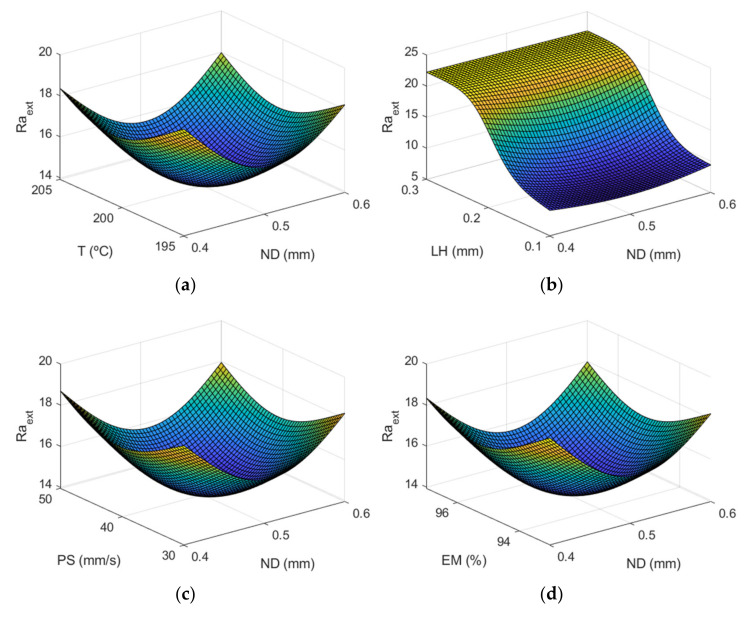

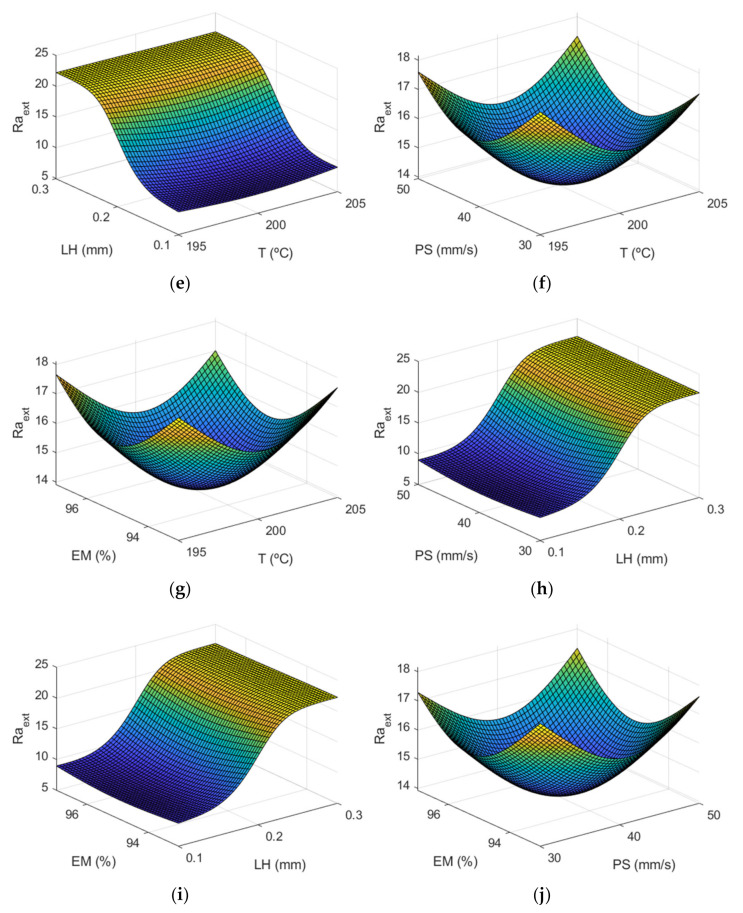
Surface response of $Ra_{ext}\left(\mu ,m\right)$ using the ANFIS for the case of the outer layer of the manufactured prototypes vs: (**a**) nozzle diameter and temperature, (**b**) nozzle diameter and layer height, (**c**) nozzle diameter and print speed, (**d**) nozzle diameter and extrusion multiplier, (**e**) temperature and layer height, (**f**) temperature and print speed, (**g**) temperature and extrusion multiplier, (**h**) layer height and print speed, (**i**) layer height and extrusion multiplier, (**j**) print speed and extrusion multiplier.

This section shows the results obtained when applying the ANFIS for modelling the above-mentioned roughness parameters. As previously mentioned, the roughness of the manufactured parts was measured inside and outside of the hemispherical cups made of PLA material that was printed using FFF technology. Figure 7 shows the surface plots of *Ra_ext_* vs different process variables.

**Figure 8:**
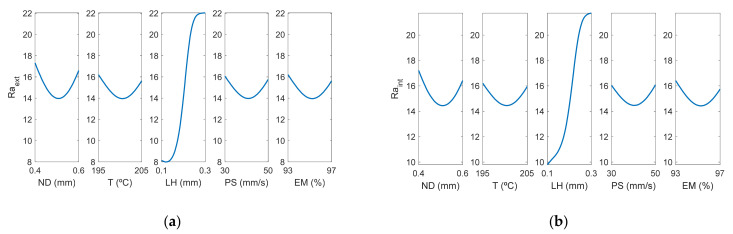
Main effects plots for (**a**) $Ra_{ext}\left(\mu ,m\right)$ and (**b**) $Ra_{int}\left(\mu ,m\right)$.

**Figure 9:**
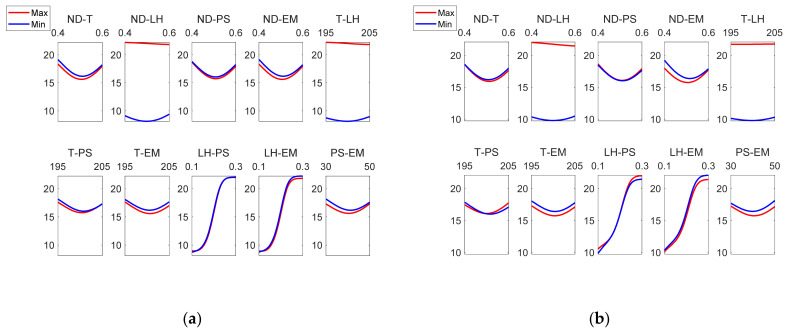
Interaction effects plots for (**a**) $Ra_{ext}\left(\mu ,m\right)$ and (**b**) $Ra_{int}\left(\mu ,m\right)$.

As can be observed in Figure 7, layer height is the most influential factor on roughness. Figure 8 corresponds to the main effects plots, while Figure 9 depicts the interaction effects plots for *Ra_ext_*.

Figure 8 and Figure 9 show that there is not a great difference between the roughness values obtained in the outer layer of the parts with respect to those obtained in the inner layer. The process parameter that had the greatest influence on the arithmetic average roughness (Ra) was the layer height, followed by the nozzle diameter. This behavior was also observed both in the roughness values obtained in the external and internal areas. In the interaction plot of ND vs LH (Figure 9a), the minimum roughness value corresponds to the situation where LH is at its → minimum value and T, EM, PS are at their central values. In this case, the model obtained by ANFIS gave a value of Ra_ext_ = 8.14 µm. Similar values were reported experimentally by Alsoufi and Elsayed [12] for nozzle diameter 0.3 mm and layer height 0.1 mm.

**Figure 10:**
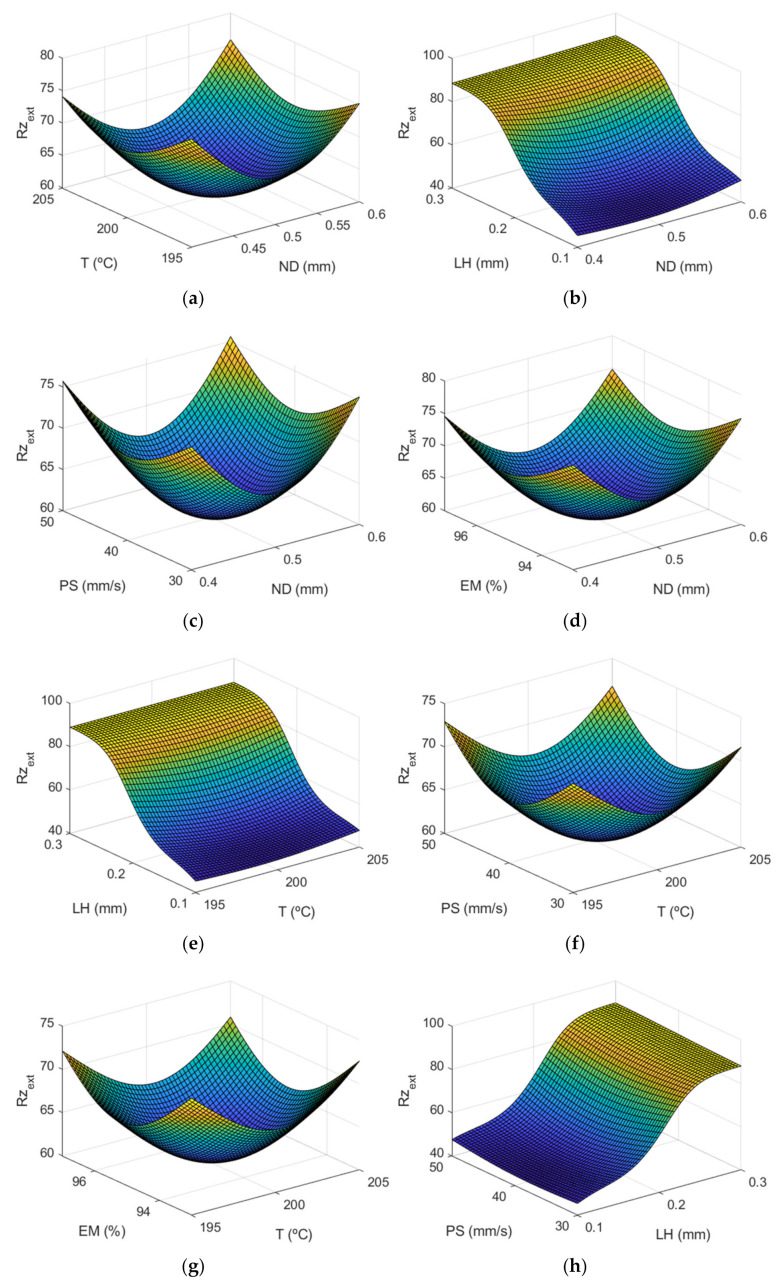

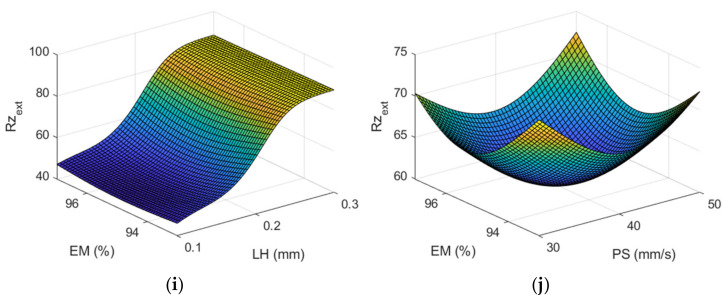
Response surface of $Rz_{ext}\left(\mu ,m\right)$ using the ANFIS for the case of the outer layer of the manufactured prototypes vs: (**a**) nozzle diameter and temperature, (**b**) nozzle diameter and layer height, (**c**) nozzle diameter and print speed, (**d**) nozzle diameter and extrusion multiplier, (**e**) temperature and layer height, (**f**) temperature and print speed, (**g**) temperature and extrusion multiplier, (**h**) layer height and print speed, (**i**) layer height and extrusion multiplier, (**j**) print speed and extrusion multiplier.

Figure 10 shows the response surface of parameter $Rz_{ext}\left(\mu ,m\right)$ using the ANFIS. A similar behavior can be observed in this figure to that obtained in the case of Ra, the layer height being the most significant factor. 

**Figure 11:**
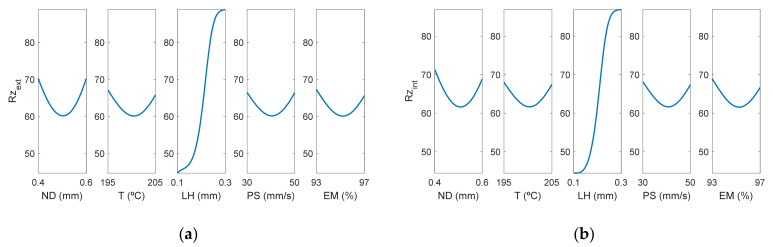
Main effects plots for (**a**) $Rz_{ext}\left(\mu ,m\right)$ and (**b**) $Rz_{int}\left(\mu ,m\right)$.

Figure 11 shows the main effect diagrams for *Rz*, the average maximum peak to valley distance of five consecutive cut-off lengths of the profile within a sampling length (l).

As can be observed in Figure 11, as could have been expected in advance, *Rz* on the internal and on the external surface presented a similar behavior.

**Figure 12:**
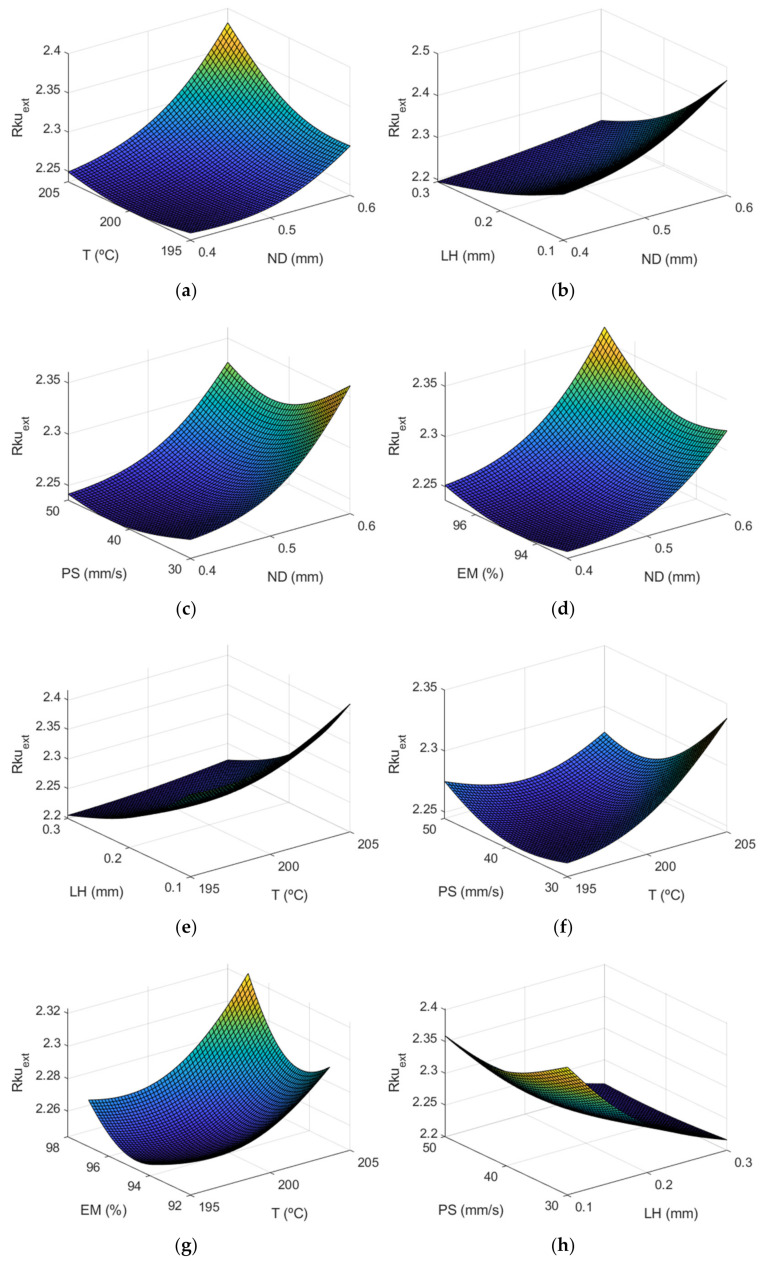

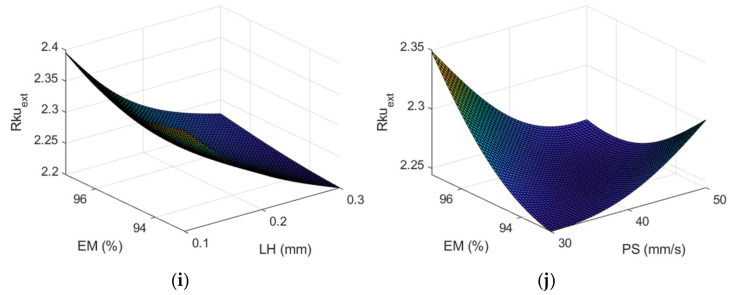
Surface response of $Rku_{ext}\left(\mu ,m\right)$ using the ANFIS for the case of the outer layer of the manufactured prototypes vs: (**a**) nozzle diameter and temperature, (**b**) nozzle diameter and layer height, (**c**) nozzle diameter and print speed, (**d**) nozzle diameter and extrusion multiplier, (**e**) temperature and layer height, (**f**) temperature and print speed, (**g**) temperature and extrusion multiplier, (**h**) layer height and print speed, (**i**) layer height and extrusion multiplier, (**j**) print speed and extrusion multiplier.

Figure 12 shows the surface response of $Rku_{ext}\left(\mu ,m\right)$ using the ANFIS for the case of the outer layer of the manufactured specimens. In this case, it can be observed that layer height influences *Rku* too.

**Figure 13:**
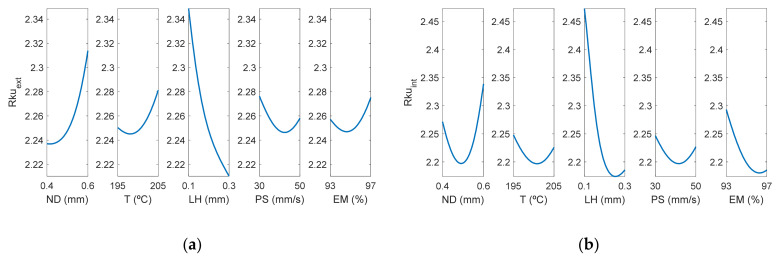
Main effects plots for (**a**) $Rku_{ext}\left(\mu ,m\right)$ and (**b**) $Rku_{int}\left(\mu ,m\right)$.

Figure 13 depicts that the most influential factor on *Rku* is layer height, followed by nozzle diameter. As can be observed in Table 3, the *Rku* parameter was lower equal or lower than 2.8 in all cases, showing that the distribution of the roughness profile was slightly rounded, to a greater extent on the external face than on the internal one. Likewise, it is shown that the parameter with the greatest influence on *Rku* was LH. In a previous work, *Rku* values around 2 were observed for low inclination angles, which decreased up to 1.7 for high inclination angles [13].

Regarding the interaction between factors, Figure 13 shows that there were differences between the external and the internal surface of the parts. Specifically, for Rku_ext_, the greatest interaction was found between PS-MS and ND-LH. On the other hand, for Rku_int_, the greatest interactions occurred between ND-EM, T-PS, LH-EM. Similar Sku values below 3 were obtained in an analogous extrusion process, direct ink writing for ceramics [37].

**Figure 14:**
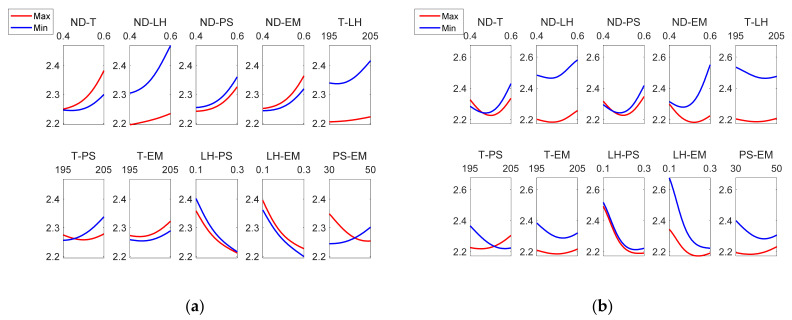
Interaction effects plots for (**a**) $Rku_{ext}\left(\mu ,m\right)$ and (**b**) $Rku_{int}\left(\mu ,m\right)$.

**Figure 15:**
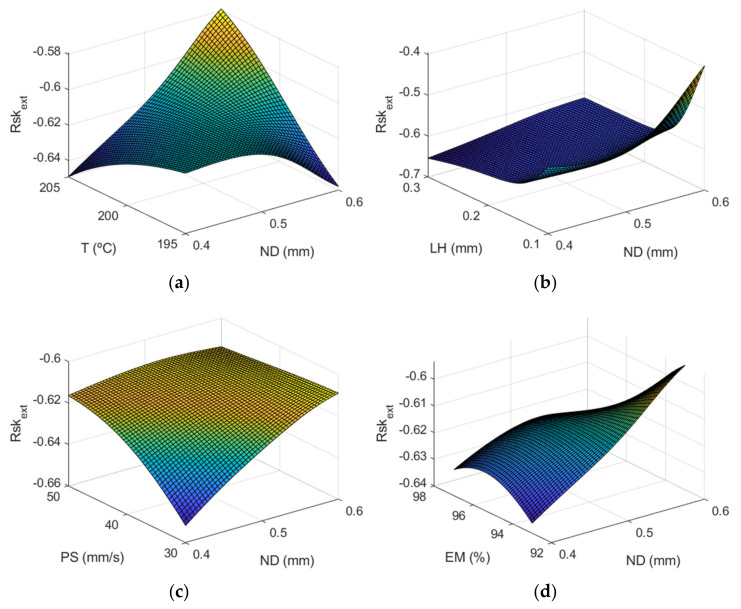

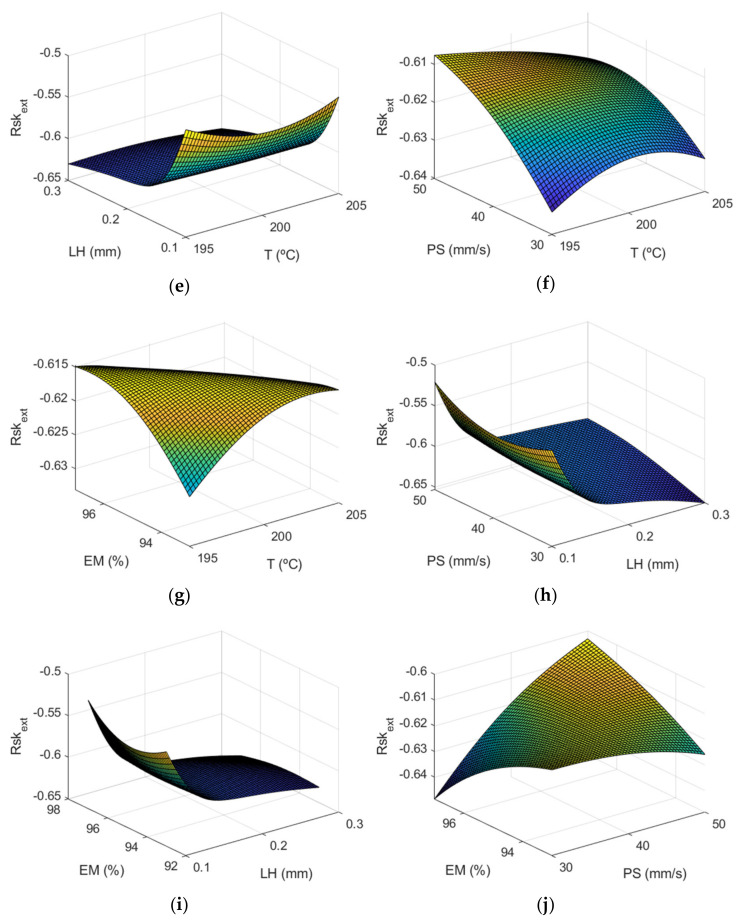
Surface response of $Rsk_{ext}\mu \left(m\right)$ using the ANFIS for the case of the outer layer of the manufactured prototypes vs: (**a**) nozzle diameter and temperature, (**b**) nozzle diameter and layer height, (c) nozzle diameter and print speed, (**d**) nozzle diameter and extrusion multiplier, (**e**) temperature and layer height, (**f**) temperature and print speed, (**g**) temperature and extrusion multiplier, (**h**) layer height and print speed, (**i**) layer height and extrusion multiplier, (**j**) print speed and extrusion multiplier.

Figure 14 shows a similar behavior regarding the interactions between *Rku_ext_* and *Rku_int_.* Figure 15 depicts the surface response of $Rsk_{ext}\left(\mu ,m\right)$ using the ANFIS for the case of the outer layer of the manufactured prototypes. Again layer height is the most influential factor on the *Rsk* parameter.

**Figure 16:**
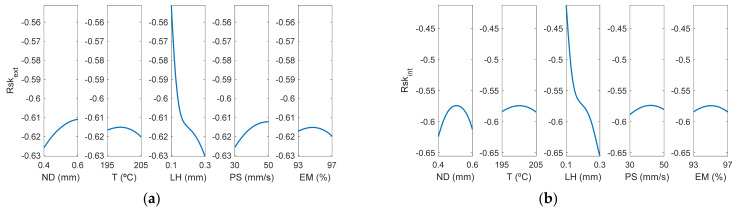
Main effects plots for (**a**) $Rsk_{ext}\mu \left(m\right)$ and (**b**) $Rsk_{int}\mu \left(m\right)$.

**Figure 17:**
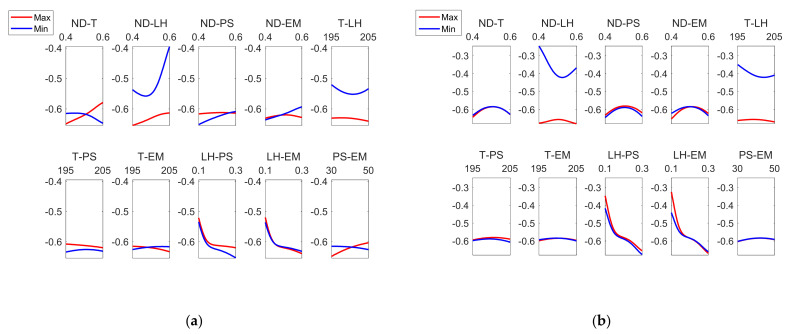
Interaction effects plots for (**a**) $Rsk_{ext}\mu \left(m\right)$ and (**b**) $Rsk_{int}\mu \left(m\right)$.

Figure 16 and Figure 17 correspond to the main effect plots and the interaction effects plots for $Rsk_{ext}\left(\mu ,m\right)$. The main effect influencing $Rsk_{ext}\left(\mu ,m\right)$ was layer height, followed by nozzle diameter and print speed.

*Rsk* took negative values in almost all experiments, as shown in Table 3, which indicates that the distribution of the valleys was more pronounced than that of the peaks in the AM parts. This is usual in extrusion 3D printing processes for vertical walls [13]. For high inclination angles, *Rsk* values increase to zero or even positive values [45]. In the present work, the lowest values of this parameter were obtained for the case of LH being 0.3 mm, and the values closest to zero were for the case of LH 0.1 mm. Likewise, it was observed that the values were more pronounced in the case of the internal face of the manufactured prototypes and also with greater variability, while in the case of the external area of the parts they were more uniform. Finally, the factor that most influenced Rsk was LH and the greatest interactions occurred for ND-LH, T-EM and PS-EM. Specifically, the ANFIS model provided an *Rsk_ex_*_t_ that was close to 0 (*Rsk_ext_* = −0.39) when LH was at its minimum value and ND at its maximum, the rest of the design parameters being at their central values. On the other hand, a value of *Rsk_int_* close to zero (*Rsk_int_* = −0.25) was obtained when ND was at its minimum value and LH at the minimum. Similar negative *Ssk* values were obtained in an analogous extrusion process, direct ink writing for ceramics [37].

## 4. Conclusions

In this research study the roughness obtained in hemispherical cups printed in PLA material with FFF technology was analyzed by using ANFIS modeling. It was found, for the range of variation of the parameters analyzed in this present study, that there is no great difference between the roughness values obtained in the outer layer of the parts with respect to those obtained in the inner layer, where the layer height, followed by the nozzle diameter, are the process parameters that have the greatest influence on both the arithmetical mean height (*Ra*) and on the greatest height of the roughness profile (*Rz*).

With regard to the kurtosis *Rku* parameter, it was shown that the distribution of the roughness profile was slightly flattened (*Rsk* < 3), to a greater extent on the external face than on the internal one, and that the layer height was the most influential parameter. Higher values were obtained for LH being 0.1 mm than for 0.3 mm. On the other hand, as a general trend the skwness parameter *Rsk* took negative values in both faces of the parts, which indicates that the distribution of the valleys was more pronounced than that of the peaks in the AM parts. The lowest values of this parameter were obtained for the case of LH being 0.3 mm and the values closest to zero were for the case of LH 0.1 mm.

The present study will help to select appropriate 3D printing conditions in order to reduce surface roughness in curved surfaces, such as those used in the manufacture of the acetabular part of the hip prostheses.

## Figures and Tables

**Table 1:** Levels of the input variables of the design of experiments (DOE).

Input Variables			Low	Center	High
ND:	Nozzle Diameter	(mm)	0.4	0.5	0.6
T:	Temperature	(°C)	195	200	205
LH:	Layer Height	(mm)	0.1	0.2	0.3
PS:	Print Speed	(mm/s)	30	40	50
EM:	Extrusion Multiplier	(%)	93	95	97

**Table 2:** Output variables (Roughness).

Outputs (Int/Ext)			
Ra (μm)	Rz (μm)	Rku	Rsk

**Table 3:** Values for the variables (ND: nozzle diameter; T: temperature; LH: layer height; PS: print speed; EM: extrusion multiplier), roughness results of the external surface and roughness results of the internal surface.

						External Roughness				Internal Roughness			
Exp.	ND	T	LH	PS	EM	Ra (μm)	Rz (μm)	Rku	Rsk	Ra (μm)	Rz (μm)	Rku	Rsk
1	0.4	195	0.1	30	97	7.58	37.57	2.319	−0.395	7.83	40.42	2.351	−0.224
2	0.6	195	0.1	30	93	8.55	40.28	2.362	−0.418	9.88	47.76	3.329	−0.502
3	0.4	205	0.1	30	93	7.86	37.96	2.262	−0.296	7.94	38.80	2.495	−0.399
4	0.6	205	0.1	30	97	9.26	48.23	2.794	−0.421	8.87	43.55	2.108	−0.167
5	0.4	195	0.3	30	93	23.37	89.74	2.130	−0.679	22.66	89.66	2.128	−0.630
6	0.6	195	0.3	30	97	21.89	86.46	2.213	−0.702	21.41	86.04	2.215	−0.693
7	0.4	205	0.3	30	97	21.20	84.42	2.298	−0.773	21.20	86.08	2.266	−0.728
8	0.6	205	0.3	30	93	21.90	91.43	2.133	−0.510	20.55	85.33	2.310	−0.720
9	0.4	195	0.1	50	93	7.92	36.77	2.380	−0.521	9.61	47.78	2.435	0.052
10	0.6	195	0.1	50	97	8.87	44.93	2.443	0.123	9.15	46.79	2.316	−0.004
11	0.4	205	0.1	50	97	8.64	41.46	2.232	−0.369	9.69	50.36	2.635	−0.009
12	0.6	205	0.1	50	93	8.90	49.41	2.580	−0.318	10.11	49.20	2.802	−0.488
13	0.4	195	0.3	50	97	22.41	91.22	2.151	−0.565	21.26	84.92	2.243	−0.694
14	0.6	195	0.3	50	93	21.55	89.08	2.224	−0.651	21.74	85.66	2.189	−0.674
15	0.4	205	0.3	50	93	22.44	88.00	2.176	−0.686	23.55	91.44	2.106	−0.654
16	0.6	205	0.3	50	97	21.77	90.76	2.194	−0.595	21.66	86.46	2.195	−0.667
17-1	0.5	200	0.2	40	95	13.94	59.94	2.297	−0.678	14.20	61.26	2.180	−0.565
17-2	0.5	200	0.2	40	95	14.18	61.67	2.239	−0.577	14.58	61.88	2.204	−0.560
17-3	0.5	200	0.2	40	95	13.84	58.85	2.207	−0.591	14.61	61.90	2.210	−0.598

## Data Availability

The data presented in this study are available upon request from the corresponding author.
